# The College of Nursing Meeting

**Published:** 1916-04-29

**Authors:** A. M. E. Bodley

**Affiliations:** Matron. Selly Oak Infirmary, Birmingham Union


*To the Editor of* The Hospital.

### The College of Nursing Meeting.

*To the Editor of* The Hospital.

Sir,—I shall be glad if you will kindly insert the following. In your issue of the 15th inst. you report that "we [Birmingham Union] have over 3,000 beds for the sick, and train over 250 nurses in the year." The actual number of beds is 2,390 and the number of nurses in training 250. Thanking you in anticipation.—Yours faithfully, A. M. E. Bodley, Matron.  
Selly Oak Infirmary, Birmingham Union,  
April 24, 1916.

